# Regional Variance‐Based Sensitivity Analysis and a Study of Regional Equifinality

**DOI:** 10.1111/risa.70346

**Published:** 2026-09-15

**Authors:** Justus Helo, Mariia Kozlova, Pamphile Roy, Julian Scott Yeomans

**Affiliations:** ^1^ LUT University Lappeenranta Finland; ^2^ Independent Researcher Vienna Austria; ^3^ Schulich School of Business York University Toronto Canada

**Keywords:** flood model, global sensitivity analysis, heterogeneity, risk analysis, Sobol' indices

## Abstract

Models in risk analysis frequently exhibit nonlinear behavior, thresholds, and regime shifts, which challenge conventional global sensitivity analysis (GSA) paradigms. Classical variance‐based methods quantify input importance over the entire output space, which can mask how these sensitivities vary across regions that are most relevant for decision making. Existing regional SA approaches, while capable of revealing such heterogeneity, are typically either qualitative or rely on unitless measures that complicate interpretation. This paper addresses these shortcomings by introducing a regional variance‐based sensitivity analysis framework that retains the interpretability of variance‐based indices while simultaneously resolving their spatial aggregation. Building on a recently developed efficient variance‐based GSA method, we extend its formulation to quantify input importance conditionally within the output‐ or input‐defined regions. The proposed approach operates directly on available input–output samples to circumvent any need for specialized experimental designs. To assess its robustness, we conduct a systematic evaluation across 16 benchmark models and nine metafunctions. We further highlight the method's ability to estimate the overall portion of the output variance explained across its regions. This property enables the study of the phenomenon of regional equifinality, which manifests as reduced overall explainability in the middle regions due to multiple combinations of inputs leading to outputs within the same region. We demonstrate the method's practical value on a canonical flood risk model, which systematically reveals substantial regime‐dependent shifts in input variable importance. The results show that this new, regional variance‐based SA enables a much deeper, decision‐relevant characterization of model behavior that bridges the existing gap between GSA and threshold‐focused risk assessment.

## 1 Introduction

For computational models to be valuable for decision making, the modeler must understand what drives the behavior of the model output (Saltelli et al. 2020). Global sensitivity analysis (GSA) is the scientific field devoted entirely to this purpose (Saltelli et al. 2008). The sole goal of GSA is to quantify the importance of input variables for the output. A wide range of mathematical tools exists within GSA to address various nuances of this goal (Iooss and Lemaître 2015; Kozlova, Lo Piano, et al. 2024; Pianosi et al. 2016).

One of the most widely adopted tools in GSA is Sobol' indices, which express the importance of input variables in terms of output variance (Tarantola et al. 2024). Sobol' indices quantify the portion of output variance explained by a given input or group of inputs (Saltelli et al. 2008). The classical estimator of Sobol' indices (Saltelli 2002; Saltelli et al. 2010) is a standard tool in major GSA software packages (Dixit and Rackauckas 2022; Iwanaga et al. 2022; Pianosi et al. 2015). Nevertheless, this estimator has several drawbacks, including (i) a complex experimental design, which limits its ability to operate on a given data set, (ii) the need for large sample sizes, and (iii) only partial compatibility with dependent input variables (Marzban and Lahmer 2016).

A recently introduced efficient method for estimating Sobol' indices addresses these drawbacks (Kozlova et al. 2025). The simple binning approach approximates Sobol' indices by (i) partitioning the input space into bins, (ii) computing the average output value within each bin, and (iii) evaluating the variance of these averages. The resulting conditional variance, when normalized by the total output variance, represents the individual effect of an input variable. Interaction effects are estimated in a similar manner by binning the corresponding multidimensional input space. This straightforward implementation negates the need for a specific experimental design and allows the method to operate directly on given data obtained from any sampling technique. These properties open new avenues for variance‐based sensitivity analysis in specific regions of the model output.

Regional sensitivity analysis (RSA) examines model sensitivity within subsets of the simulation data. It originated in two‐region analyses that separated outcomes into desired and undesired, or more generally behavioral and nonbehavioral, cases, an approach often referred to as Monte Carlo filtering or Hornberger–Spear–Young (HSY) generalized sensitivity analysis (Beven 2018; Hornberger and Spear 1981). The two regions are then compared visually, through conditional and unconditional distributions, or quantitatively, using distance statistics such as the Kolmogorov–Smirnov statistic. This logic was later extended to RSA with multiple output regions, often defined by quantiles, while retaining the same visual and distribution‐distance comparisons (Pianosi et al. 2016). Attempts to apply variance‐based sensitivity analysis to regions of model output exist, but the classical estimator faces multiple problems, including computational expense, the dependence of independent inputs when conditioned on output, and the inability to judge whether a full variance of the output is explained when only first‐order and total indices are computed (Marrel and Chabridon 2021).

This paper introduces a regional variance‐based sensitivity analysis method that extends the efficient simple binning approach (Kozlova et al. 2025) to regional analysis. The contribution is threefold. First, we conduct an extensive evaluation of the binning approach in comparison with the classical estimator of Sobol' indices and more efficient surrogate‐modeling‐based indices to assess its reliability on small samples. Second, we examine the revealed phenomenon of regional equifinality, which is defined as the loss of output variance explainability in middle regions. We also show that surrogate‐modeling‐based indices are unable to capture regional equifinality because they rely on a global approximation of the model response. Third, we illustrate the approach through an application to a flood model, which yields new insights when compared to the existing sensitivity analyses of the model.

The new regional variance‐based sensitivity analysis method is application‐agnostic and can be applied to any computational model or input–output data set. However, its value is particularly pronounced for models that exhibit heterogeneous behavior (Kozlova, Moss, et al. 2024), as it reveals how the sensitivity profile changes across regions of the input and output space. The method is especially relevant to models in the risk analysis domain that are generally characterized by thresholds and regime‐switching behavior.

The remainder of the paper is structured as follows. Section 2 introduces the general logic of variance‐based sensitivity analysis and presents the classical polynomial chaos expansion (PCE), simple binning estimators, and their comparative evaluation across multiple benchmark functions. Section 3 introduces the algorithm for the regional variance‐based sensitivity analysis method. Section 4 investigates the phenomenon of regional equifinality on a simple additive model and several benchmark functions. Section 5 demonstrates the method on a flood model case study. The paper concludes with a brief discussion and final remarks.

## 2 Existing Variance‐Based Sensitivity Analysis Methods

Variance‐based sensitivity analysis is based on the idea of variance decomposition, where all effects are expressed as portions of the output variance (Sobol' 1993). Individual, or first‐order, effects are defined as

(1) $$S_{i}=\frac{\mathrm{Var}\left[E(Y|X_{i})\right]}{\mathrm{Var}(Y)}.$$

Interaction, or second‐order, effects refer to the additional contribution beyond individual effects arising from the interaction between two input variables:

(2) $$S_{ij}=\frac{\mathrm{Var}\left[E(Y|X_{i},X_{j})\right]}{\mathrm{Var}(Y)}-S_{i}-S_{j}.$$

Also, total effects are formulated in the following way and, in essence, represent the sum of all‐order effects that one input variable has with others (Homma and Saltelli 1996):

(3) $$S_{T_{i}}=1-\frac{\mathrm{Var}\left[E(Y|X_{∼i})\right]}{\mathrm{Var}(Y)}.$$

As an example, for a two‐factor model, the total effects of $X_{1}$ and $X_{2}$ are

(4) $$S_{T_{1}}=S_{1}+S_{12},\;S_{T_{2}}=S_{2}+S_{12}.$$

The sum of total indices is therefore

(5) $$S_{T_{1}}+S_{T_{2}}=S_{1}+S_{2}+2S_{12}.$$

The doubled interaction term implies that the sum of total indices equals 1 only in the case of a fully additive model, where interaction effects are zero. When interactions are present, the sum of first‐order indices is less than 1, while the sum of total indices exceeds 1.

To address this issue, some researchers proposed variance‐based indices based on the fair Shapley allocation rule (Owen 2014), to ensure that the indices always sum to 1 regardless of the presence of interactions. For a two‐factor model, this formulation takes the form:

(6) $$S_{1}^{\mathrm{Sh}}=S_{1}+\frac{1}{2}S_{12},\;S_{2}^{\mathrm{Sh}}=S_{2}+\frac{1}{2}S_{12}.$$

The sum of Shapley‐based indices is therefore

(7) $$S_{1}^{\mathrm{Sh}}+S_{2}^{\mathrm{Sh}}=S_{1}+S_{2}+S_{12}.$$

### 2.1 Classical Sobol' Indices Estimator

The classical estimators for Sobol' indices include first‐order (Saltelli 2002) and total‐effect indices (Homma and Saltelli 1996). Second‐order (and higher‐order) indices are typically obtained using extensions of the same Monte Carlo framework (Saltelli 2002; Saltelli et al. 2008).

The estimator requires a specific design of experiments (DoE), in which several Monte Carlo simulations need to be run to produce several input–output data sets (or matrices), Algorithm 1.

ALGORITHM 1 **Pseudocode for the classical estimation of first‐order Sobol' indices.**

**Table:** 

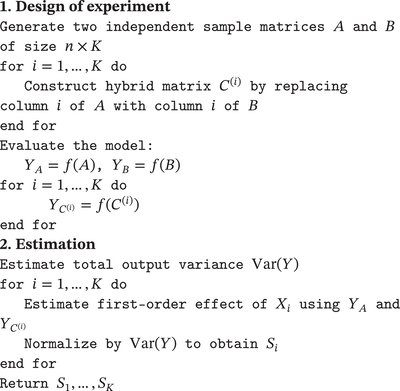

For first‐order and total indices, the estimator relies on two base sample matrices and one additional hybrid matrix per input, resulting in a total of N=n(K+2) model evaluations (Saltelli 2002). Quasi‐Monte Carlo (QMC) is recommended for use with the classical estimator, which requires n to be a power of 2.

### 2.2 Efficient PCE Estimator

The PCE is a surrogate model approach, which can be used to estimate classical Sobol' indices with a low computational cost (Sudret 2008). It approximates the given model using a set of orthogonal polynomial functions with respect to the input variables distributions. The coefficients are estimated using regression methods such as Ordinary Least Squares (OLS). Sparse alternatives such as Least‐Angle Regression (LARS) (Blatman and Sudret 2011) are commonly used in high‐dimensional settings to estimate the Sobol' indices.

The model output Y=M(X), where $X=\{X_{1},\cdots ,X_{K}\}$ is a K‐dimensional random vector, can be approximated by a truncated PCE:

(8) $$Y\approx \hat{Y}=\sum_{\alpha \in A}y_{\alpha }\Psi_{\alpha }\left(X\right),$$

where A is a set of multi‐indices defining the truncation strategy, $y_{\alpha }$ are the coefficients, and $\Psi_{\alpha }\left(X\right)$ are multivariate polynomials orthonormal with respect to the joint probability density function of X.

The coefficients $y_{\alpha }$ are calculated by OLS over a DoE (Algorithm 2).

ALGORITHM 2 **Pseudocode for the PCE‐based estimation of first‐order Sobol' indices.**

**Table:** 

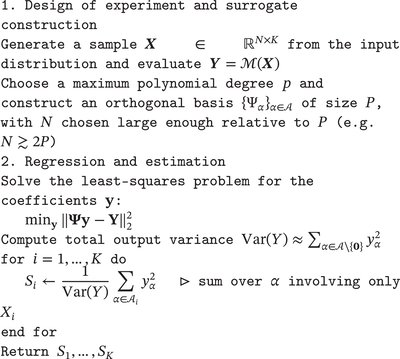

### 2.3 Efficient Simple Binning Estimator

Unlike the classical estimator, the simple binning approach operates on a single input–output data set and does not require a dedicated sampling design (Algorithm 3) (Kozlova et al. 2025).

ALGORITHM 3 **Pseudocode for the simple binning estimation of first‐order Sobol' indices.**

**Table:** 

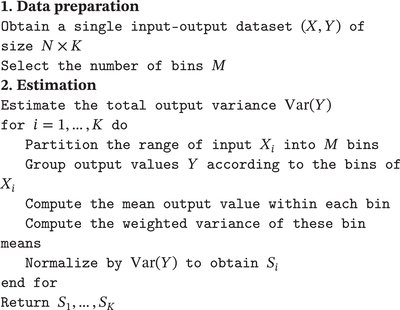

For input $X_{i}$, let $B_{im}$ denote bin m, $N_{im}$ the number of observations in that bin, ${\bar{Y}}_{im}$ the mean output in that bin, and $\bar{Y}$ the overall output mean. The first‐order effect estimator of the simple binning approach is the weighted variance of bin‐wise conditional output means divided by the total output variance,

(9) $${\hat{S}}_{i}=\frac{\sum_{m=1}^{M_{i}}N_{im}{\left({\bar{Y}}_{im}-\bar{Y}\right)}^{2}}{\sum_{n=1}^{N}{\left(Y^{\left(n\right)}-\bar{Y}\right)}^{2}}.$$

The second‐order is obtained similarly, accounting only for joint pairwise effects using two‐dimensional bins and subtracting the corresponding first‐order effects (equation 2).

In addition to first‐ and second‐order effects, the simple binning method computes so‐called combined indices, which in practice correspond to Shapley effects up to second order (Equation 6). Unlike total effects, combined indices enable a diagnostic conservation property: when the combined indices sum to one, the decomposition accounts for the full output variance.

All indices are estimated from a single input–output data set. The optimal number of bins, determined experimentally, is automatically selected based on the data set size, while the number of bins used for second‐order estimation is taken as the square root of the number used for first‐order effects (Kozlova et al. 2025). The method is agnostic to the sampling technique. Beyond simple random and quasi‐random sampling, it has been applied to full‐factorial design (García Pérez et al. 2024) and empirical data (Kuokkanen 2026).

The simple binning method is open‐sourced (Roy and Kozlova 2024) and has been successfully applied across a wide range of domains, including business (Myers et al. 2026; Walzer et al. 2026), environmental (Jeong et al. 2025; Zaikova et al. 2025), and engineering applications (Havia et al. 2025; Pesonen et al. 2025; Saari et al. 2024).

### 2.4 Performance Comparison

#### 2.4.1 Testing Setup

The three estimators, classical (Saltelli 2002), PCE (Sudret 2008), and simple binning (Kozlova et al. 2025) are tested side by side on the 16 benchmark models (Azzini and Rosati 2022; Kucherenko et al. 2011) and six metafunctions (Becker 2020) shown in Table 1. The benchmark functions provide a variety of test cases for varying sensitivity index structures and they follow Kucherenko's taxonomy of difficulty (Kucherenko et al. 2011). Metafunctions are an approach that uses a randomized set of basis functions that are used to build first‐, second‐, and third‐order effects (Becker 2020). The list of benchmark models and basis functions is found in Table 1.

**TABLE 1:** Benchmark test functions and their mathematical formulations.

Function	Mathematical expression
**A1**	$f\left(x\right)=\sum_{j=1}^{n}{\left(-1\right)}^{j}\prod_{k=1}^{j}x_{k},\;U{\left(0,1\right)}^{n}$
**B1**	$f\left(x\right)=\prod_{j=1}^{n}\frac{n-x_{j}}{n-0.5},\;U{\left(0,1\right)}^{n}$
**C1**	$f\left(x\right)=2^{n}\prod_{j=1}^{n}x_{j},\;U{\left(0,1\right)}^{n}$
**C2**	$f\left(x\right)=\prod_{j=1}^{n}\left|4x_{j}-2\right|,\;U{\left(0,1\right)}^{n}$
**F1**	$f\left(x\right)=10x_{1}+0.5x_{2}^{3},\;U{\left(-5,5\right)}^{2}$
**F2**	$f\left(x\right)=2x_{1}-x_{2}^{2},\;U{\left(-5,5\right)}^{2}$
**F3**	$f\left(x\right)=x_{1}^{2}+x_{2}^{4}+x_{1}x_{2}+x_{2}x_{3}^{4},\;U{\left(-4,4\right)}^{3}$
**F4**	$f\left(x\right)=0.2e^{x_{1}-3}+2.2\left|x_{2}\right|+1.3x_{2}^{6}-2x_{2}^{2}-0.5x_{2}^{4}-0.5x_{1}^{4}+2.5x_{1}^{2}+0.7x_{1}^{3}+\frac{3}{{\left(8x_{1}-2\right)}^{2}+{\left(5x_{2}-3\right)}^{2}+1}+\sin\left(5x_{1}\right)\cos\left(3x_{1}^{2}\right),\;U{\left(-1,1\right)}^{2}$
**F5**	$f\left(x\right)=\frac{1}{2^{n}}\prod_{j=1}^{n}\left(3x_{j}^{2}+1,\;U{\left(0,1\right)}^{n}\right)$
**F6**	$f\left(x\right)=\exp\left(x_{1}+2x_{2}\right),\;U{\left(0,1\right)}^{n}$
**G**	$f\left(x\right)=\prod_{j=1}^{n}\frac{|4x_{j}-2|+a_{j}}{1+a_{j}},\;\text{where}\;a_{1}=a_{2}=0,\;\text{and}\;a_{j>2}=6.52,\;U{\left(0,1\right)}^{n}$
G*	$f\left(x\right)=\prod_{j=1}^{n}\frac{\left(1+\alpha_{j}\right){\left|2(x_{j}+\delta_{j}-I\left\lfloor x_{j}+\delta_{j}\right\rfloor )-1\right|}^{\alpha_{j}}+a_{j}}{1+a_{j}},a_{j}\neq -1,\delta_{j}\in \left[0,1\right],\alpha_{j}>0,\;U{\left(0,1\right)}^{n}$
**Hartmann 6‐D**	$f\left(x\right)=-\sum_{i=1}^{4}\alpha_{i}\exp\left(-\sum_{k=1}^{6}A_{i,k}{\left(x_{k}-P_{i,k}\right)}^{2}\right),\;U{\left(0,1\right)}^{6},$ $\text{with}\;\alpha ={\left[1.0,1.2,3.0,3.2\right]}^{\prime },$ $A=\left[\begin{array}{cccccc} 10 & 3 & 17 & 3.50 & 1.7 & 8\\ 0.05 & 10 & 17 & 0.1 & 8 & 14\\ 3 & 3.5 & 1.7 & 10 & 17 & 8\\ 17 & 8 & 0.05 & 10 & 0.1 & 14 \end{array}\right]$, $P=10^{-4}\left[\begin{array}{cccccc} 1312 & 1696 & 5569 & 124 & 8283 & 5886\\ 2329 & 4135 & 8307 & 3736 & 1004 & 9991\\ 2348 & 1451 & 3522 & 2883 & 3047 & 6650\\ 4047 & 8828 & 8732 & 5743 & 1091 & 381 \end{array}\right]$
**Ishigami**	$f\left(x\right)=\sin\left(x_{1}\right)+7\sin^{2}\left(x_{2}\right)+0.1x_{3}^{4}\sin\left(x_{1}\right),\;U{\left(-\pi ,\pi \right)}^{3}$
**Kriging**	$f\left(x\right)=1+\exp\left(-2\left({\left(x_{1}-1\right)}^{2}+x_{2}^{2}\right)-0.5\left(x_{3}^{2}+x_{4}^{2}\right)\right)+\exp\left(-2\left(x_{1}^{2}+{\left(x_{2}-1\right)}^{2}\right)-0.5\left(x_{3}^{2}+x_{4}^{2}\right)\right),\;U{\left(0,1\right)}^{4}$
**Owen**	$f\left(x\right)=\prod_{j=1}^{n}\left[\mu_{j}+\tau_{j}\;g_{j}\left(x_{j}\right)\right],\text{with}\;g_{j}\left(x_{j}\right)=\sqrt{12}\;\left(x_{j}-0.5\right),n=6,\;\mu_{j}=1,\;\tau_{j}=\left[1,1,0.5,0.5,0.25,0.25\right],\;U{\left(0,1\right)}^{6}$
**Metafunctions**	$f\left(x\right)=\sum_{j=1}^{d}\alpha_{j}g_{u_{j}}\left(x_{j}\right)+\sum_{i=1}^{\left\lfloor 0.5d\right\rfloor }\beta_{i}\left(g_{V_{i,1}}g_{V_{i,2}}\right)+\sum_{i=1}^{\left\lfloor 0.2d\right\rfloor }\gamma_{i}\left(g_{W_{i,1}}g_{W_{i,2}}g_{W_{i,3}}\right),\;U{\left(0,1\right)}^{d}$ *Component functions* $g_{k}\left(x\right)$: $g_{1}=x$; $g_{2}=x^{2}$; $g_{3}=x^{3}$; $g_{4}=\frac{e^{x}-1}{e-1}$; $g_{5}=\frac{\sin(2\pi x)}{2}+\frac{1}{2}$; $g_{6}=I_{x\geq 0.5}$; $g_{7}=0$; $g_{8}=4{\left(x-0.5\right)}^{2}$; $g_{9}=\frac{1}{\left(10-\frac{1}{1.1}\right)\left(x+0.1\right)}-0.1$

For each model and method, we compare two sampling strategies, simple random and QMC, and three sample sizes, approximately $10^{2}$, $10^{3}$, and $10^{4}$. They are calculated with N=n(2K+2) as required by the SAlib Python library for QMC sampling (SAlib.sample.sobol) and estimating the Sobol' indices (SAlib.analyze.sobol). The exact sample sizes for each function vary depending on the number of input variables due to the sampling requirements of the classical estimator and QMC methods. For each pair of sampling strategies (simple versus quasi‐random), the sample size is held constant to enable direct comparison. PCE is implemented with the OpenTURNS Python library (Baudin et al. 2016) and simple binning method is implemented with the SimDec Python library and its simdec.sensitivity_indices function (Simulation‐Decomposition 2026).

Thus, for each test function, six experiments are conducted (two sampling strategies by three sampling sizes), with each result presented as a separate comparison box plot. Each experiment is repeated 999 times to assess the precision of the resulting indices; accordingly, each box represents 999 realizations of the same index.

#### 2.4.2 Test Results

A comprehensive catalog of all of the test results is presented in Appendix A. This section provides a brief summary of the main patterns, illustrated through selected examples.

**FIGURE 1:**
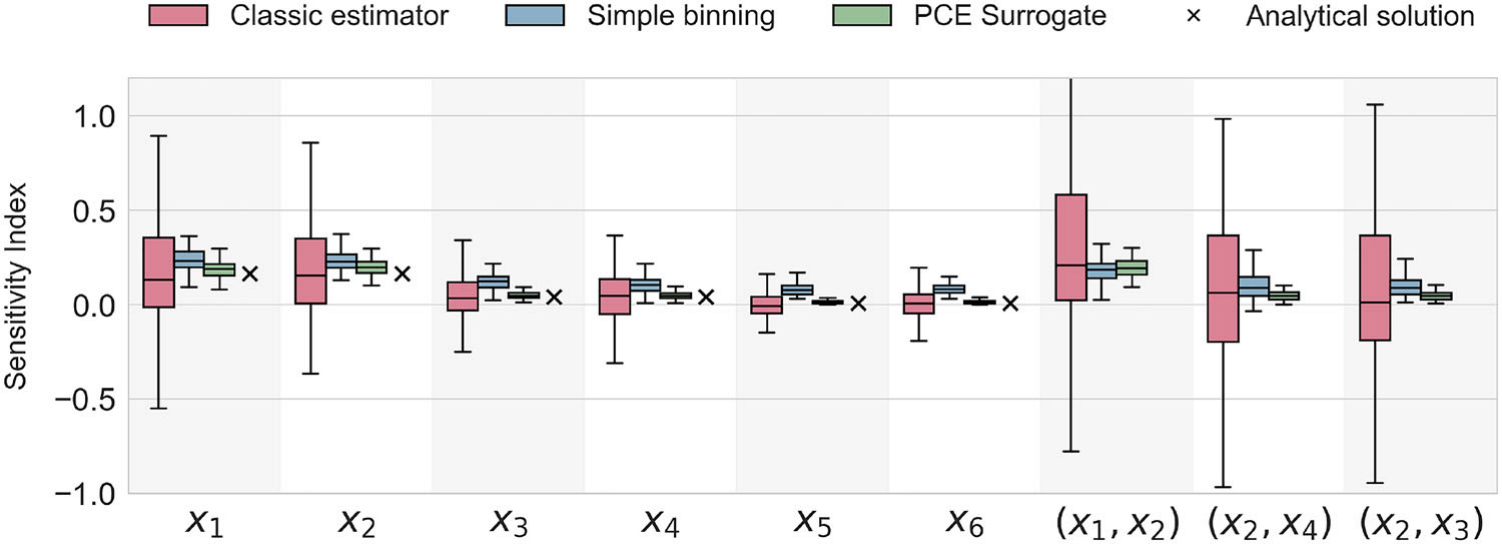
Sensitivity indices for the Owen function, simple random sampling, N=112.

The key discovery is that the PCE and simple binning approach outperform the classical estimator by reducing the noise in the estimates by several times. This pattern is consistent across all functions, sample sizes, and sampling techniques. Notably, PCE produces reasonably precise estimates even for sample sizes as small as $10^{2}$ obtained via simple random sampling, whereas the classical estimator exhibits such high variability that individual estimates cannot be considered reliable. Simple binning exhibits slightly higher variance compared to PCE with sample sizes of $10^{2}$ but reaches nearly as accurate estimates with the $10^{3}$ samples (Figure 1).

**FIGURE 2:**
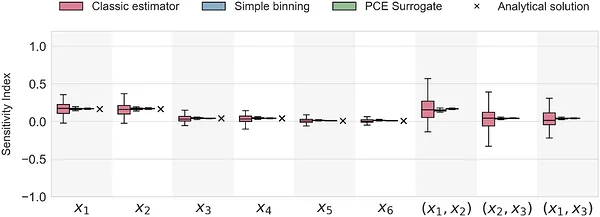
Sensitivity indices for the Owen function, simple random sampling, N=1792.

**FIGURE 3:**
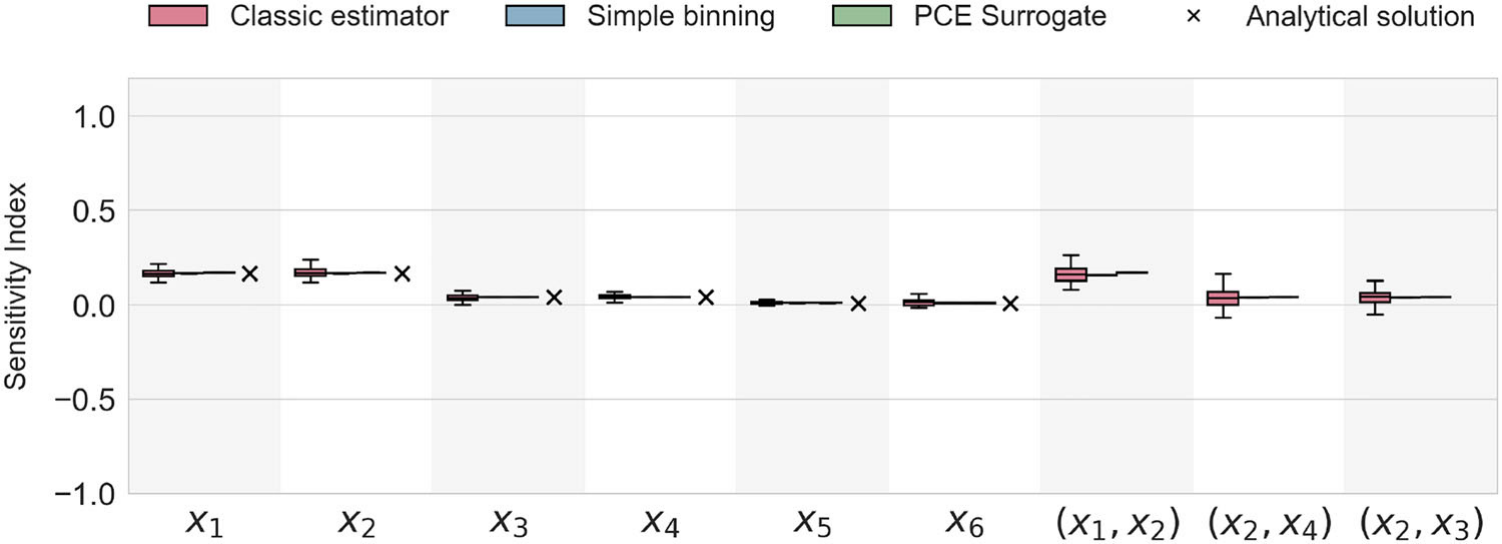
Sensitivity indices for the Owen function, quasi‐Monte Carlo, N=14,336.

Of major practical significance, the precision of PCE and simple binning indices obtained from a simple random sample of size 1792 (Figure 2) exceeds that of the classical estimator computed from a QMC sample that is eight times larger (Figure 3). This computational relationship holds across all other functions.

One drawback of the simple binning method, visible, for example, in the Owen function (Yun et al. 2017) in Figure 1, is that it has introduced a slight bias by shifting the estimates up/down relative to the analytical solution due to the binning mechanics. However, this shift is minor, does not distort the ranking of variable importance, and diminishes rapidly with larger sample sizes (Figure 2).

**FIGURE 4:**
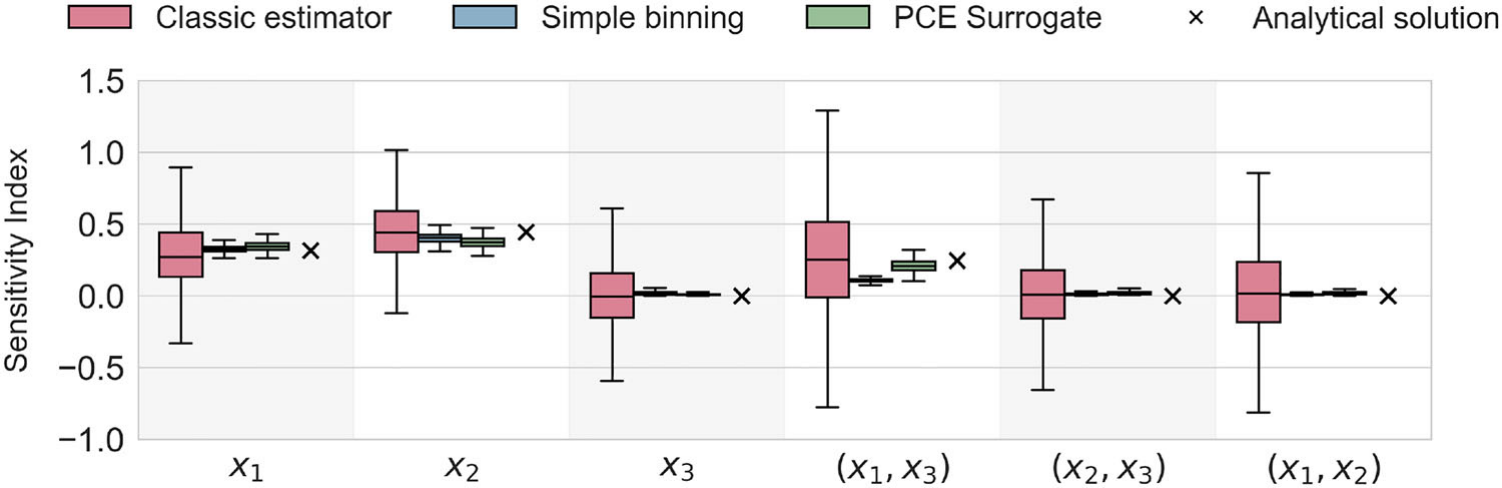
Sensitivity indices for the Ishigami function, quasi‐Monte Carlo, N=128.

An example of effects underestimated by the simple binning approach can be observed in the highly nonlinear Ishigami function (Ishigami and Homma 1990) (Figure 4). Second‐order effects are particularly prone to this, as the square root of the number of bins is used in their estimation. Consequently, for models with pronounced cyclical behavior or local irregularities, larger sample sizes are required to adequately incorporate these nonlinearities into the sensitivity indices.

**FIGURE 5:**
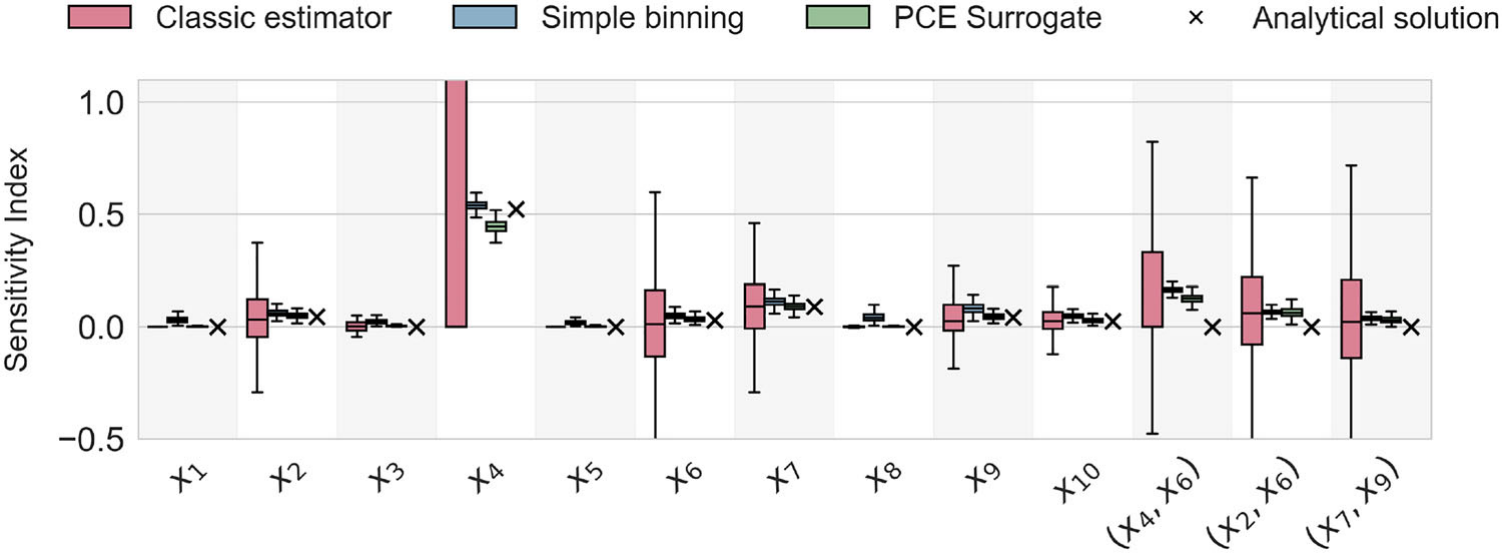
Sensitivity indices for metafunction 2, quasi‐Monte Carlo, N=176.

One peculiar feature of the classical estimator is its tendency to cancel out noise for zero indices. It is especially pronounced for metafunctions (Becker 2020) (Figure 5). This same finding is shared with PCE since coefficients fit with OLS are deterministic. This property is, of course, absent in simple binning estimates due to fundamental algorithmic differences. Nevertheless, the precision of simple binning estimates around zero remains acceptable.

Overall, PCE and the simple binning method demonstrate several times higher precision of sensitivity estimates than the classical estimator. They able to produce meaningful indices even for samples as small as $10^{2}$ obtained with basic sampling strategies. The drawbacks of binning do not materially distort the performance of the simple binning method. These characteristics, together with their ability to operate on a given data set, make PCE and simple binning suitable candidates for RSA.

## 3 A New Method for Regional Variance‐Based Sensitivity Analysis

The procedure for the proposed regional variance‐based sensitivity analysis consists of computing variance‐based sensitivity indices using the simple binning approach within user‐defined regions. As in classical regional analysis, the regions can be defined either behaviorally (e.g., profitable vs. nonprofitable) or based on quantiles, and can be constructed for either output variables or inputs. For completeness, the algorithm of the analysis is provided in Algorithm 4.

ALGORITHM 4 **Pseudocode for regional variance‐based sensitivity analysis.**

**Table:** 

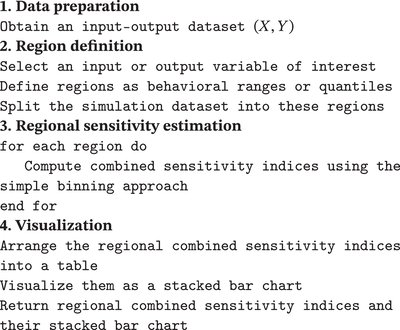

The analysis yields regional sensitivity indices expressed as shares of explained output variance that account for first‐ and second‐order effects. The corresponding stacked bar chart highlights not only the relative importance of individual input variables, but also the total explained variance reflected in the overall bar height (i.e., the sum of the indices).

The binning algorithm is open‐source, requires only an input–output data set, and is available in Python, R, and Matlab with a single function call (Simulation‐Decomposition 2026).

## 4 A Study of Regional Equifinality

Compared with conventional distribution‐based RSA, where regional importance is assessed by comparing conditional and unconditional input distributions, variance‐based RSA permits an examination of how much of the output variance remains explainable within each output region. A reduced influence of individual inputs in middle regions has been observed before in conventional RSA (Spear et al. 2020) and interpreted as equifinality. Variance‐based RSA makes it possible to inspect this phenomenon in greater detail.

As an illustrative example, consider the simplest possible model, Y=A+B, in which A and B are independent binary variables taking values in {0,1}. The output can take only three values, Y∈{0,1,2}. The extreme outcomes are uniquely determined: Y=0 can occur only when A=0 and B=0, while Y=2 can occur only when A=1 and B=1. In contrast, the middle outcome Y=1 can be generated by two different input configurations, either (A,B)=(0,1) or (A,B)=(1,0). Thus, the mapping from inputs to output is no longer one‐to‐one in the middle region. If this region is considered in isolation, the observed output does not reveal which of the two compensating input configurations produced it; one would need an additional latent variable indicating which pair is active. Since no such variable exists in the original model, the individual effects of A and B become difficult to distinguish within this region.

Equivalently, the state A=0 is compatible with both Y=0 and Y=1, while A=1 is compatible with both Y=1 and Y=2; the same is true for B. Conditioning on the middle output region therefore weakens the discriminatory power of each input considered separately. What matters in this region is not the isolated value of A or B, but their compensating combination. This illustrates the basic mechanism of equifinality: the same output region can be reached through multiple input configurations, so the overall sum of regional sensitivity indices decreases when the region is analyzed in isolation.

For the numerical experiment, both inputs, A and B, are allowed to vary independently and uniformly over the range [1,10]. The model is simulated 10,000 times, and the output sample is divided into 10 quantile regions, each containing 1000 data points.

**FIGURE 6:**
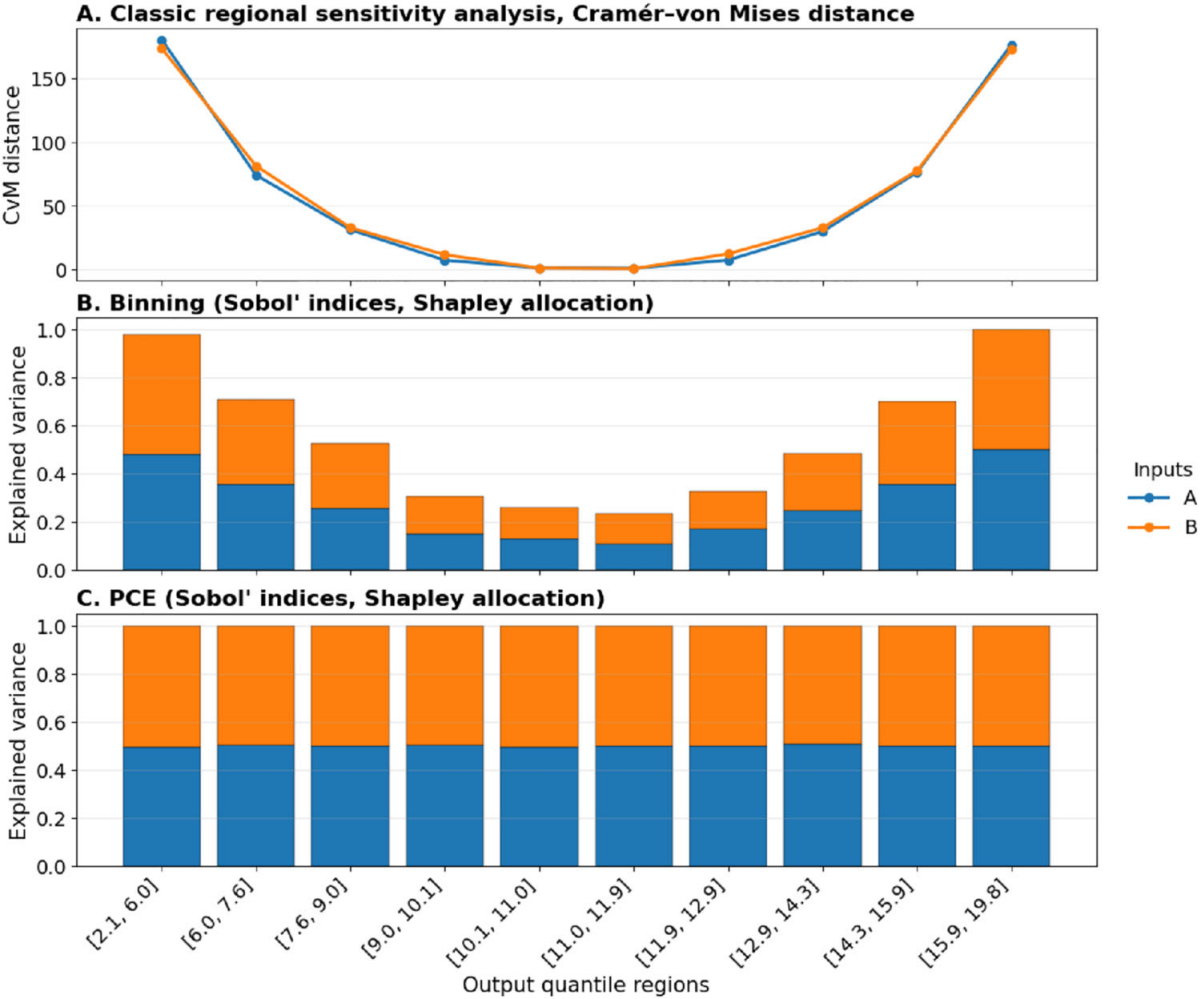
Regional sensitivity analysis of a simple additive model with independent inputs using different approaches.

Figure 6A shows the pattern commonly observed in conventional RSA: the apparent influence of each input decreases in the middle output regions. Figure 6B shows the same phenomenon using variance‐based RSA. Here, the explained output variance attributed to each input is allocated according to the Shapley rule and can therefore be summed, or stacked, across inputs. If the stacked bar approaches 1, the variance of the output within that region is almost fully explained by the inputs under the regional decomposition. Bars that remain substantially below 1 indicate a loss of regional explainability. This loss corresponds to equifinality: middle output regions can be reached through compensating combinations of A and B, making the contribution of each input harder to identify when the region is analyzed in isolation.

Figure 6C repeats the analysis using PCE‐based indices, with the interaction term allocated according to the same Shapley rule. Unlike the variance‐based RSA results in Figure 6B, the PCE‐based indices sum to one in every region. This occurs because PCE fits a polynomial metamodel within each region and decomposes the variance of that fitted representation. The procedure normalizes the regional variance decomposition and removes the very signal of interest: the decline in the explainable share of output variance because multiple compensating input configurations produce similar outputs. Transformation‐based PCE workflows for dependent inputs led to the same behavior, with index sums equal to one across regions. Therefore, while PCE remains valuable for global surrogate modeling, it is not suitable for detecting regional equifinality.

Importantly, the presence of equifinality within output regions should not be interpreted as a failure to explain the variance of the model as a whole. For the additive model, Y=A+B, the global sensitivity results in Table 2 show that both the simple‐binning and PCE‐based indices explain nearly all output variance. The two inputs contribute almost equally, and the second‐order term is essentially zero. The loss of explainability observed in the middle regions is therefore a regional phenomenon caused by conditioning on the output, not a property of the global model.

**TABLE 2:** Global variance‐based sensitivity indices for the additive model with independent inputs.

Index	Simple binning	PCE
$S_{A}$	0.49	0.50
$S_{B}$	0.50	0.50
$S_{AB}$	0.00	0.00
Sum	0.99	1.00

**FIGURE 7:**
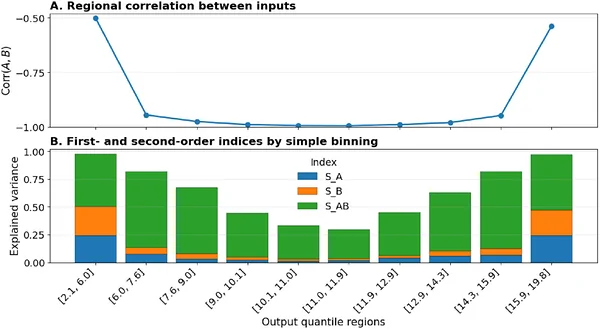
Correlation of independent inputs in regions of the output and break down of the sensitivity effects.

Within output regions, the two independent inputs of the additive model become negatively dependent: the same values of Y can be produced either by low A and high B, or by high A and low B. Figure 7A confirms this induced dependence, with the regional correlation decreasing from about −0.5 in the edge regions to nearly −1 in the middle regions. As also shown in earlier work (Kozlova et al. 2025), this negative dependence gives rise to a nonzero interaction terms, shown in Figure 7B. Interestingly, the second‐order contribution accounts for about half of the output variance in the edge regions and becomes almost the only contribution present in the middle regions. This occurs despite the fact that the underlying model is additive and has no global interaction effect, as shown in Table 2.

**FIGURE 8:**
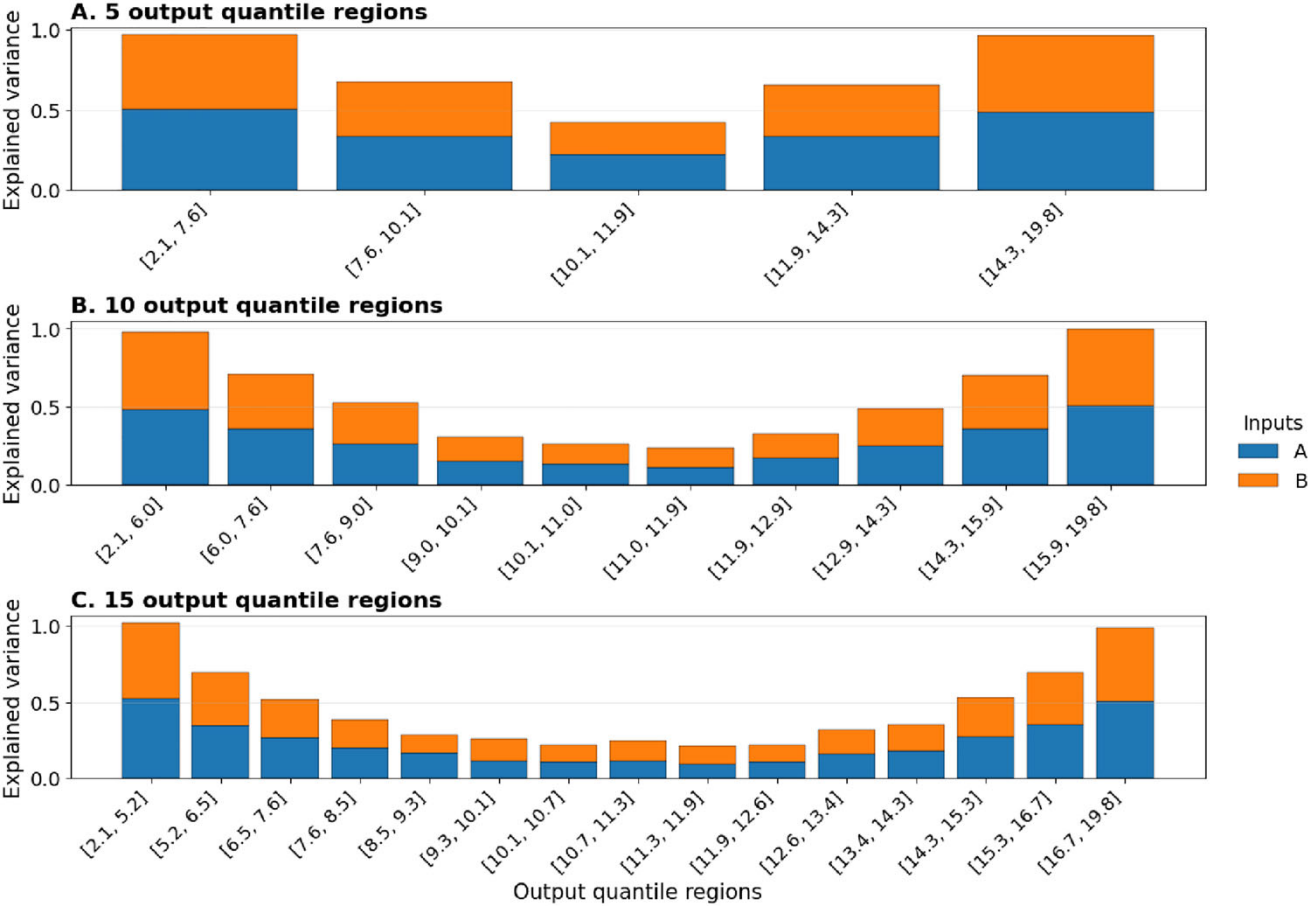
Persistence of equifinality across different numbers of output regions.

**FIGURE 9:**
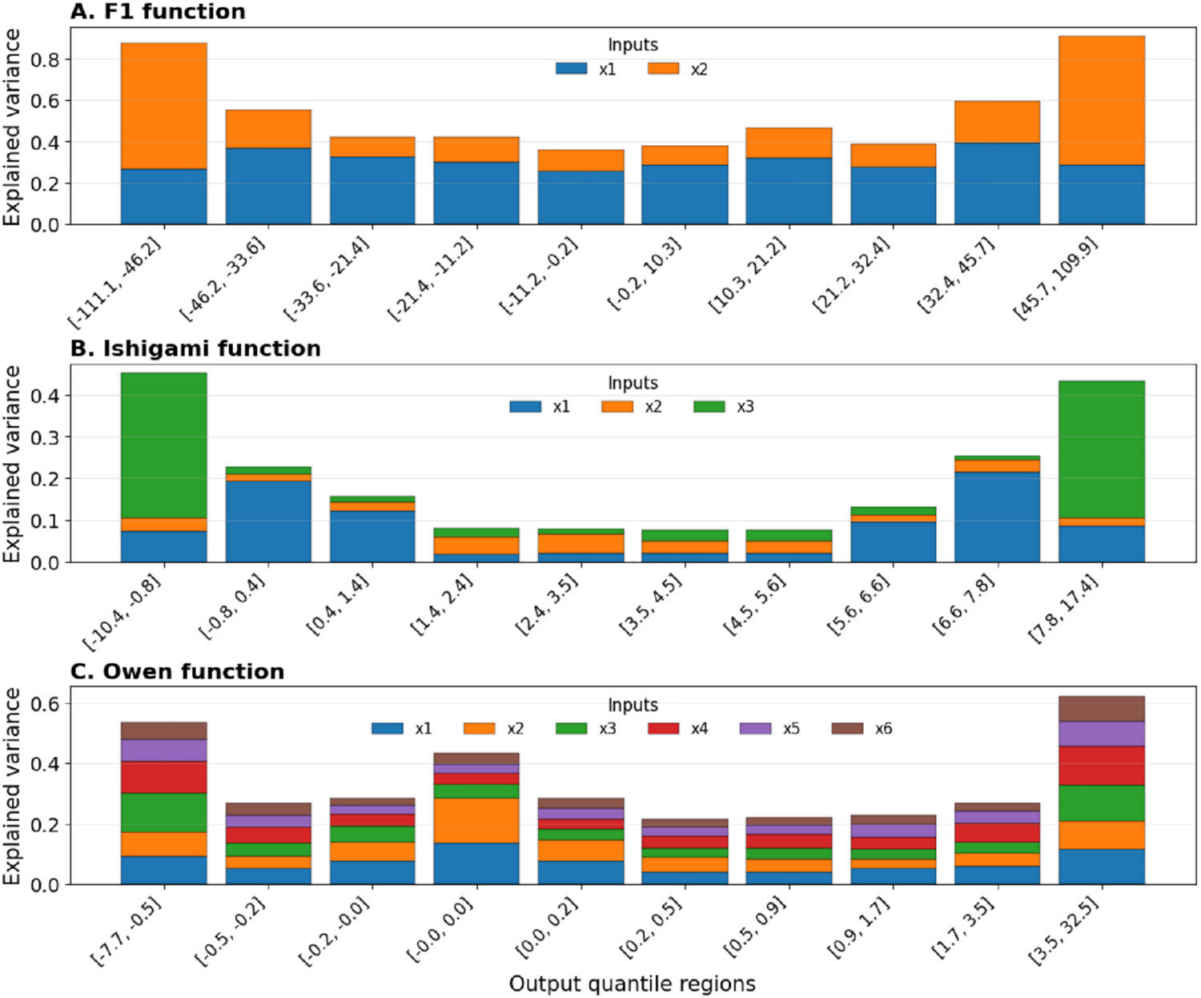
Equifinality across different benchmark models.

Equifinality persists across different numbers of output regions (Figure 8), and across different model structures (Figure 9). More complex models show that equifinality does not necessarily follow the typical U‐shaped pattern. This is especially clear for the Owen function, where the regional pattern is shaped by a local peak in the model behavior. Variance‐based RSA makes it possible to examine regional equifinality together with the changing influence of individual inputs across the output space. In the Ishigami function, for example, the edge regions are dominated by $x_{3}$ and have the highest overall explainability; $x_{1}$ becomes more influential away from the edges, while the middle regions show a stronger role of $x_{2}$ and the largest drop in overall explainability.

**FIGURE 10:**
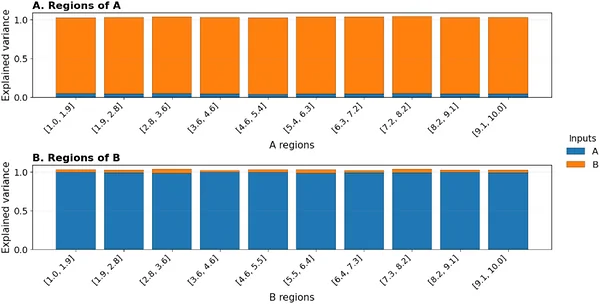
Absence of equifinality in regions of input space.

A situation in which equifinality does not occur is when regions are defined by conditioning on the inputs rather than on the output. Figure 10 illustrates this for the additive model, Y=A+B. When the analysis is conditioned on the input space, each region corresponds to a full model simulation over a truncated input distribution. Unlike output‐conditioned regions, these regions do not merge multiple compensating input configurations into the same output band. The relationship between the remaining input variation and the output is therefore preserved, and the regional sensitivity indices can account for the full output variance within each region.

Overall, variance‐based RSA reframes equifinality as a regional property of the input–output mapping: not a failure of the model to explain variance globally, but a loss of local explainability caused by conditioning on output regions that can be reached through multiple compensating input configurations.

## 5 Case Study

### 5.1 Flood Model

This section will examine a simplified flood model that has been analyzed extensively in several previous studies (Baudin et al. 2016; de Rocquigny 2006; De Rocquigny 2012; Iooss and Lemaître 2015; Limbourg and De Rocquigny 2010). The model's aim is to assess the relationship between its uncertain hydraulic and geometric inputs to three outputs: the river water depth, the overflow above the dyke, and an associated total cost.

The following input variables are used to describe the model:

- Q: flow rate ($m^{3}/s$)
- $K_{s}$: Strickler coefficient ($m^{1/3}/s$)
- $Z_{v}$: downstream bed elevation (m)
- $Z_{m}$: upstream bed elevation (m)
- B: river width (m)
- L: river reach length (m)
- $Z_{b}$: bank elevation (m)
- $H_{d}$: dyke height (m)

The elevation of the dyke crest is

(10) $$Z_{d}=Z_{b}+H_{d}.$$

Assuming a mild slope and approximately uniform flow, the river slope is modeled as

(11) $$\alpha =\frac{Z_{m}-Z_{v}}{L},\;Z_{m}\geq Z_{v}.$$

Using a Manning–Strickler‐type relation for a large rectangular section, the water depth is written as

(12) $$H={\left(\frac{Q}{K_{s}B\sqrt{\alpha }}\right)}^{0.6}.$$

The water‐surface elevation is then

(13) $$Z_{c}=Z_{v}+H.$$

Flooding occurs when the water surface rises above the dyke crest. The corresponding overflow variable (in meters) is

(14) $$S=Z_{c}-Z_{d}.$$

Hence, a flood event is defined by

(15) S>0.

The flood probability can therefore be written as

(16) $$P_{f}=P\left(S>0\right).$$

#### 5.1.1 Cost Model

The total cost is represented as the sum of dyke construction cost and flood cost, expressed in million euros (Iooss and Lemaître 2015; OpenTURNS 2026):

(17) $$C=C_{d}+C_{s}.$$

The dyke cost is modeled by

(18) $$C_{d}=\left\{\begin{array}{cc} \frac{8}{20}, & \text{if}\;H_{d}<8,\\ \frac{H_{d}}{20}, & \text{otherwise}. \end{array}\right.$$

The flood‐related cost is modeled as

(19) $$C_{s}=\left\{\begin{array}{cc} 1-0.8\exp\;\left(-\frac{1000}{S^{4}}\right), & \text{if}\;S<0,\\ 1, & \text{otherwise}. \end{array}\right.$$

A flood (S>0) incurs a fixed cost of 1 million euros. In the absence of a flood (S≤0), the cost represents the operational and maintenance expenses of the infrastructure, which rise as the safety margin approaches zero and settle at a baseline level as the margin widens.

#### 5.1.2 Uncertain Inputs

The model treats the inputs as mutually independent random variables. A common specification is

$$Q∼\text{Gumbel}\left(1013,558\right),\;K_{s}∼N\left(30,7.5^{2}\right)\;\text{truncated to}\;K_{s}>0,$$

$$Z_{v}∼U\left(49,51\right),\;Z_{m}∼U\left(54,56\right),$$

B∼Triangular(295,300,305),L∼Triangular(4990,5000,5010),

$$Z_{b}∼\text{Triangular}\left(55,55.5,56\right).$$

Dyke height, depending on the version of the model used, is either deterministic, or uniformly distributed from 7 to 9, or two scenarios are checked:

(20) $$H_{d}∼U\left(2,4\right)\;\text{(low-dyke scenario),}$$

or

(21) $$H_{d}∼U\left(7,9\right)\;\text{(high-dyke scenario).}$$

### 5.2 Existing Sensitivity Analyses

Existing sensitivity analyses of the flood model include first‐order and total Sobol' indices computed globally for the entire model (OpenTURNS 2026) and visualization of effects with the parallel coordinates plot (Baudin et al. 2016). Various other global importance measures have been tested on this model as well (Iooss and Lemaître 2015).

**FIGURE 11:**
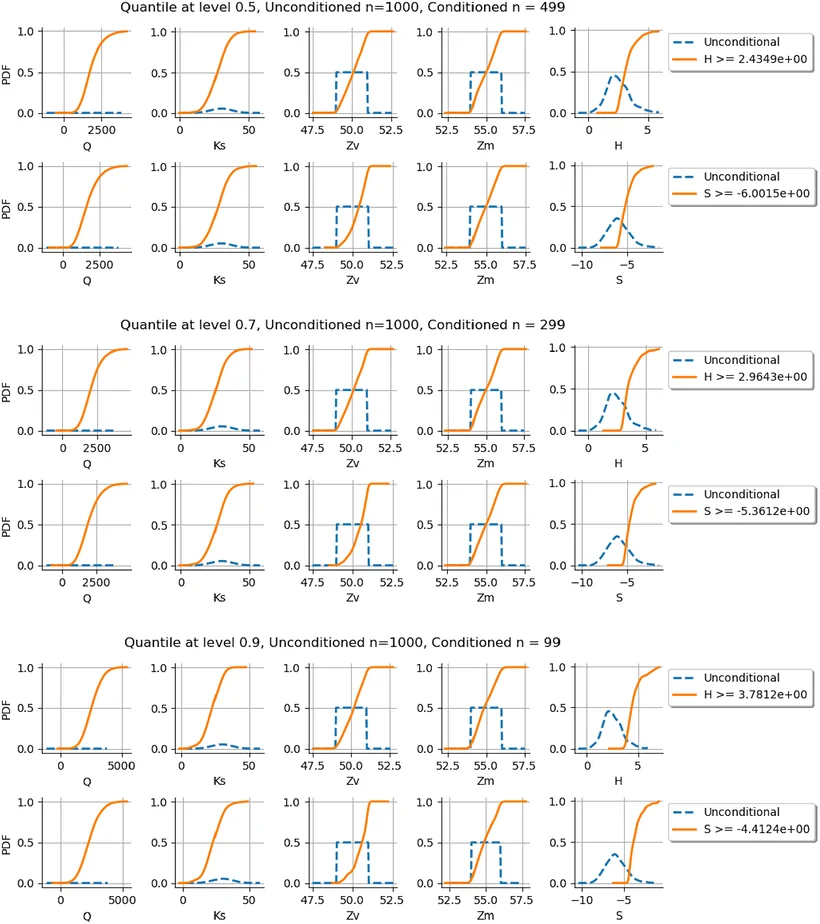
Sensitivity plots across 0.5, 0.7, and 0.9 quantile levels (Baudin 2026).

However, the flood model is characterized by varying importance across different regions of its output space. Such model behavior provoked further explorations. A visual RSA compared conditional and unconditional output distributions across regions of the outputs (Baudin 2026). Figure 11 presents the sensitivity plots for the 0.5, 0.7, and 0.9 quantile levels. This approach confirmed that input importance varies substantially across regions. However, it relies on large numbers of graphical comparisons and lacks formal aggregation into quantitative indices.

**FIGURE 12:**
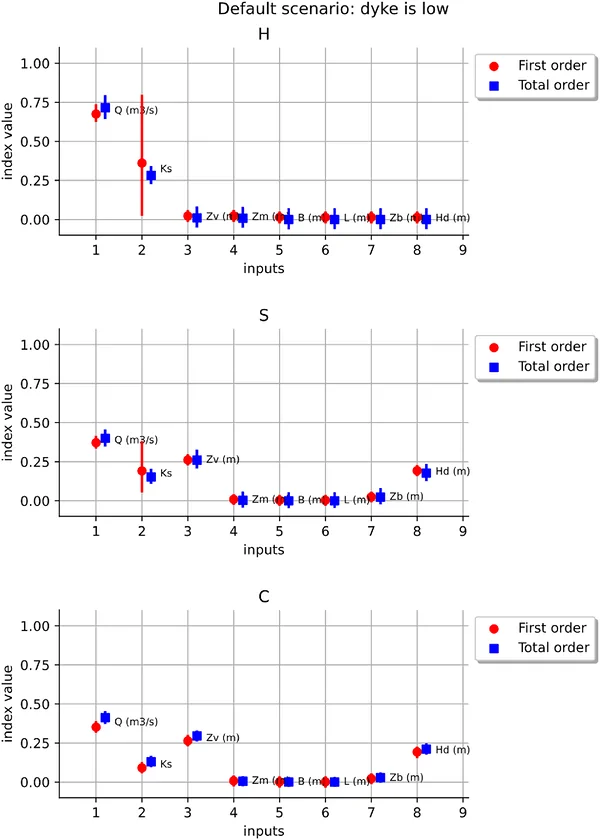
First‐order and total sensitivity indices for the low‐dyke scenario H (OpenTURNS 2026).

**FIGURE 13:**
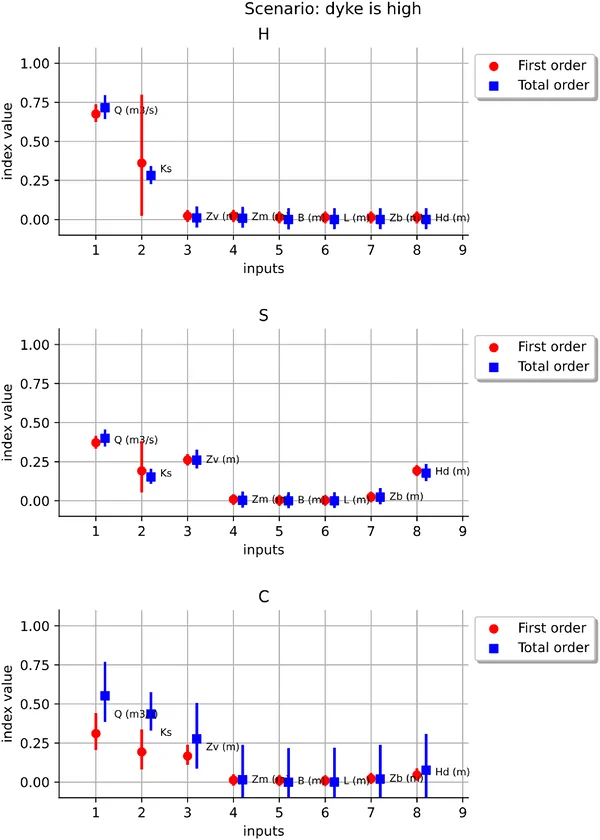
First‐order and total sensitivity indices for high‐dyke scenario H (OpenTURNS 2026).

An alternative sensitivity analysis of the model was obtained through input regime switching, that is, computing and comparing sensitivities for low‐ and high‐dyke height (OpenTURNS 2026). The two regimes look similar for H and S, but produce very different sensitivity profiles for cost C. Downstream height $Z_{v}$ is more impactful in the low‐dyke scenario than smoothness of the riverbed $K_{s}$ (Figure 12), and vice versa in the high‐dyke scenario (Figure 13).

This approach relies on manually splitting the regions and analyzing the distributions qualitatively, rather than on formally aggregated indices.

These existing analyses clearly demonstrate regional sensitivity heterogeneity of the flood model, while leaving room for a more systematic and quantitative representation.

### 5.3 New Results by Regional Variance‐Based Sensitivity Analysis

The version of the model formulated in Section 5.1 but with dyke height $H_{d}$ scenarios merged into single continuous range U(2,9), was evaluated $10^{5}$ times using simple random sampling. Regional variance‐based sensitivity indices for the three outputs, water depth H, overflow S, and cost C, were computed using 10 quantiles, each containing $10^{4}$ simulation points. Their values are aggregated via a stacked bar chart.

**FIGURE 14:**
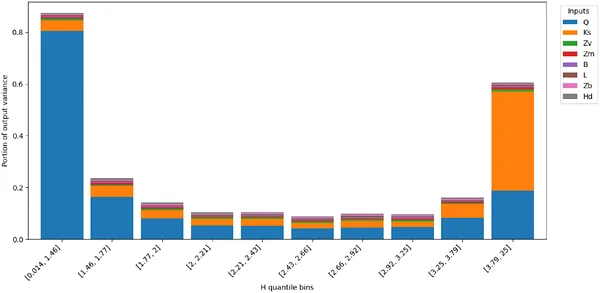
Combined sensitivity indices for regions of water depth H.

The variance of water depth H is predominantly driven by the flow rate Q, with a stronger influence at the lowest depth values (Figure 14). At higher depth levels, the variance is primarily governed by the riverbed smoothness $K_{s}$. Similar conclusions were obtained from the previous visual analysis of 15 individual plots for H (Figure 11). The new analysis, however, gives more detail quantile‐wise. For example, the sensitivity structure appears relatively uniform in the intermediate range, and the dominance of $K_{s}$ only appears in the top quantile (Figure 14). The sums of combined indices for all but the first and last quantiles remain below approximately 0.2 manifesting equifinality.

**FIGURE 15:**
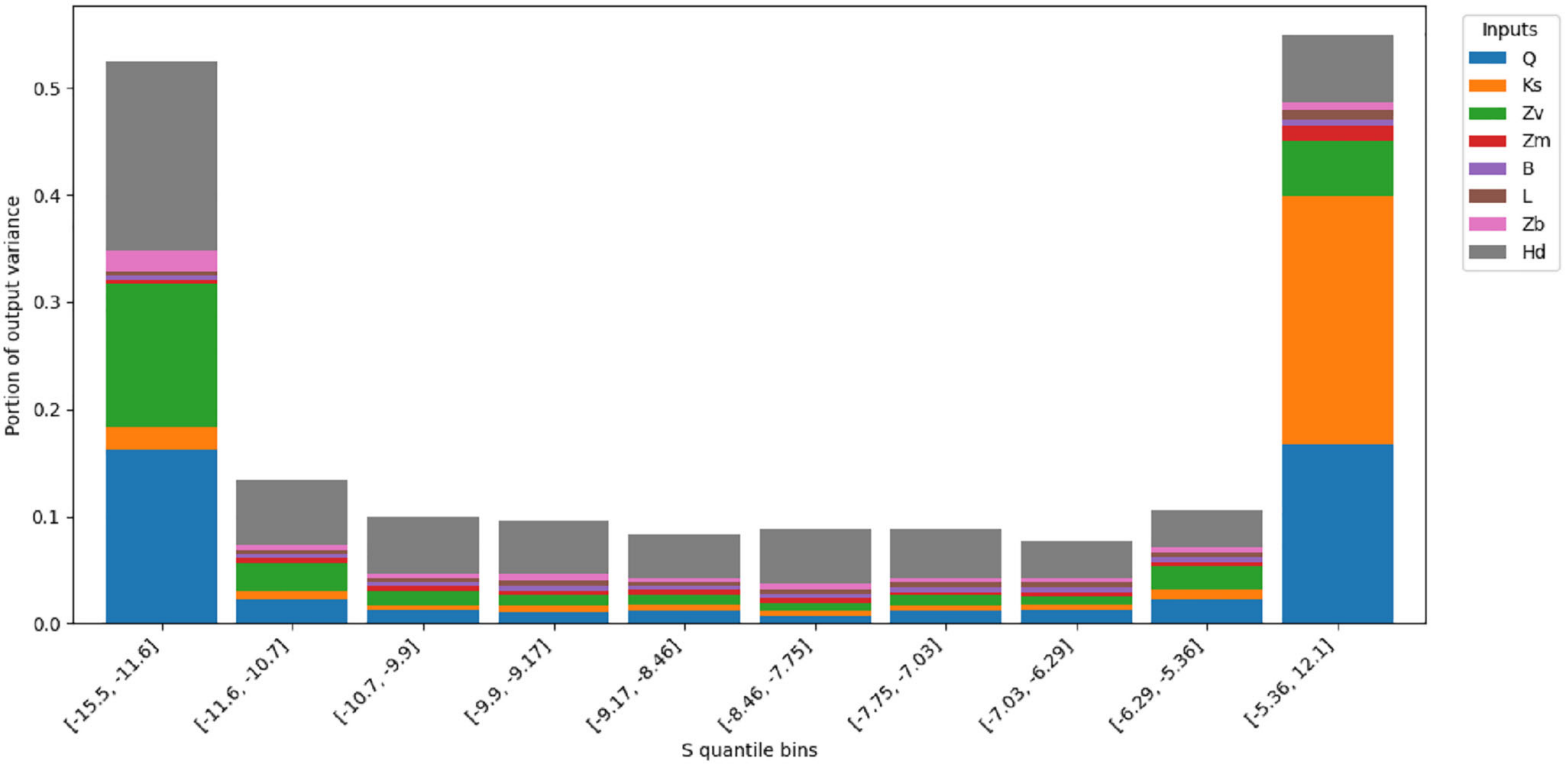
Combined sensitivity indices for regions of overflow S.

The overflow S exhibits a more complex sensitivity profile (Figure 15). In the first quantile, corresponding to deeply negative overflow values, three variables contribute at comparable levels: the flow rate Q, downstream bed elevation $Z_{v}$, and dyke height $H_{d}$. In the last quantile, which captures flood events, the riverbed smoothness $K_{s}$ becomes the dominant factor, reducing the influence of the other variables. Across the intermediate quantiles, the dyke height emerges as the primary driver. As with the previous output H, the sums of the indices in the middle quantiles remain below approximately 0.2.

Interestingly, there are differences with the previous analysis. A visual inspection of the 15 plots for S (Figure 11) would seem to imply that $Z_{v}$ dominates in all quantiles followed by the influences of Q and $K_{s}$ (Baudin 2026). However, our analysis tells a different story in which $Z_{v}$ shares a roughly equal contribution with Q and $K_{s}$ only in the first quantile, playing only a minor role thereafter. Consequently, this approach provides a more direct and quantitatively explicit reading of regional sensitivities.

**FIGURE 16:**
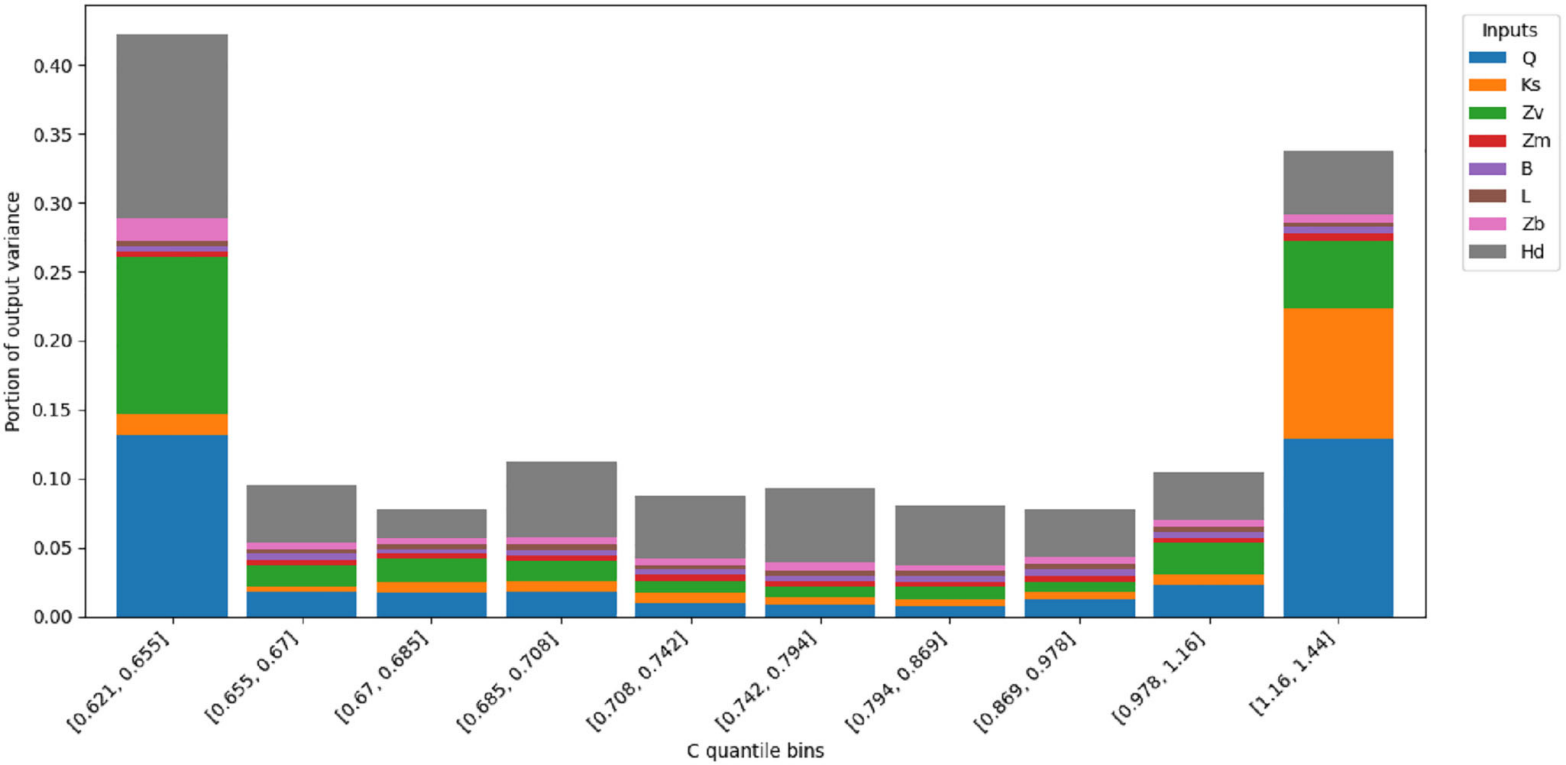
Combined sensitivity indices for regions of costs C.

The sensitivity profile for the costs C, largely mirrors that of the overflow S, with one notable exception: in the last quantile, the flow rate Q becomes more dominant than the riverbed smoothness $K_{s}$ (Figure 16).

An input‐RSA analysis can be performed that builds upon the earlier analysis of for two scenarios of $H_{d}$, low (2, 4) (Figure 12) and high (7, 9) (Figure 13). The now continuous range of dyke height $H_{d}$ that encompasses both scenarios is broken down into 10 quantiles, and the sensitivities to all three outputs are computed. The three lower quantiles correspond to the previous low scenario and the three upper ones to the high scenario.

**FIGURE 17:**
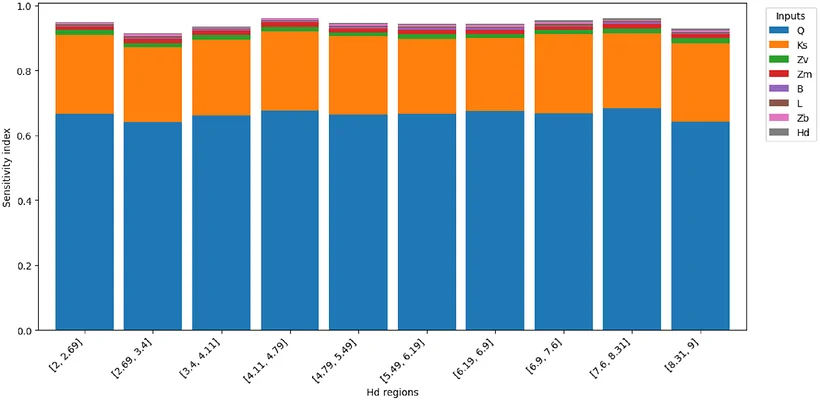
Combined sensitivity indices for the river depth H in the regions of $H_{d}$.

**FIGURE 18:**
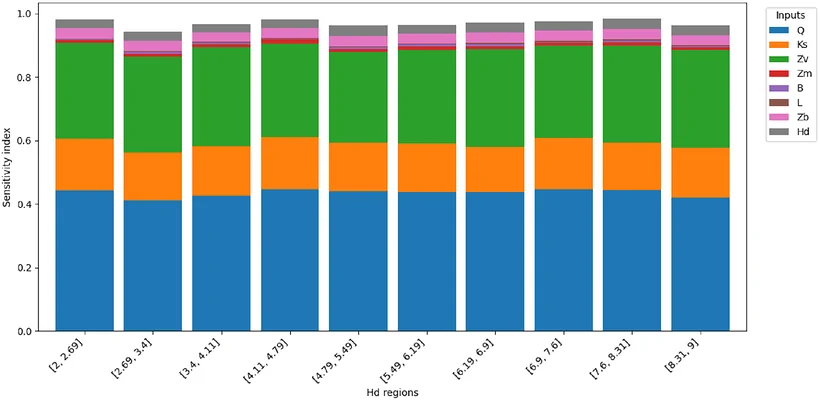
Combined sensitivity indices for the overflow S in the regions of $H_{d}$.

The sensitivity profiles of river depth H and overflow S appear stable (Figures 17 and 18), which is consistent with the previous findings (Figures 12 and 13).

As noted in Section 4, in such cases where the input space is partitioned rather than the output space, equifinality does not happen: each region corresponds to a separate full simulation and, thus, the sums of combined indices across all quantiles approach one.

**FIGURE 19:**
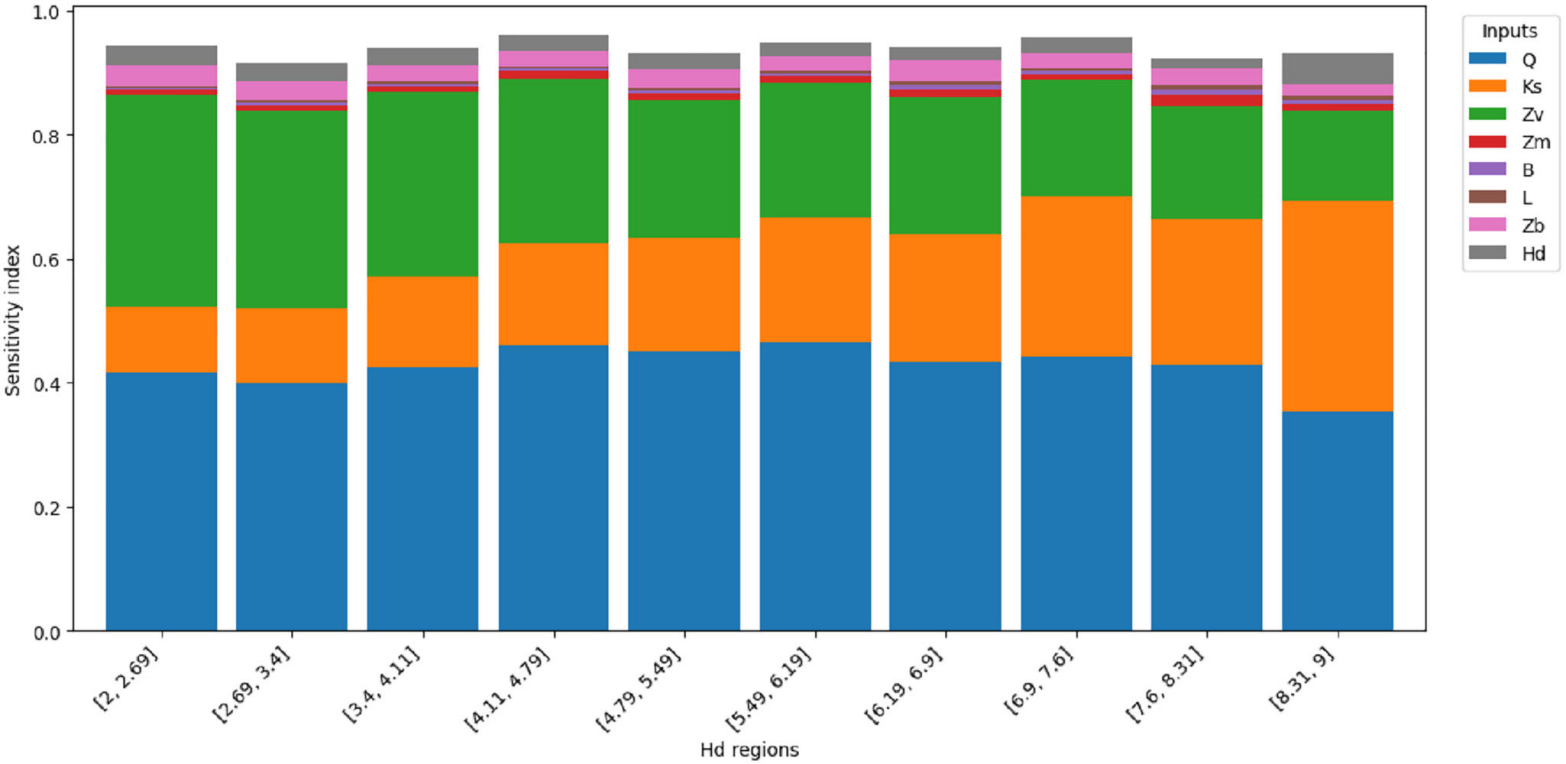
Combined sensitivity indices for the cost C in the regions of $H_{d}$.

When costs C are considered as the output, some variation in sensitivity emerges across different ranges of dyke height (Figure 19). As dyke height increases, the influence of $Z_{v}$ gradually decreases, while the influence of $K_{s}$ increases correspondingly. The same conclusions can be drawn from the previous analysis. The impact of Q remains relatively stable across low and mid ranges of dyke height, but begins to decline in the upper quantiles. The contribution of dyke height, itself, to the variance of costs is slightly higher in the last quantile which suggests increasing marginal costs with larger construction size. This is a new finding as the previous analysis merged the three upper quantiles into a single high scenario and the increased influence of $H_{d}$ in the last quantile was diluted. Furthermore, the earlier approach would generally require 10 plots to display indices for 10 quantiles, which would make the graphics harder to perceive. Conversely, the single plot of Figure 19 displays the indices of all quantiles right next to each other thereby permitting direct comparisons.

**FIGURE 20:**
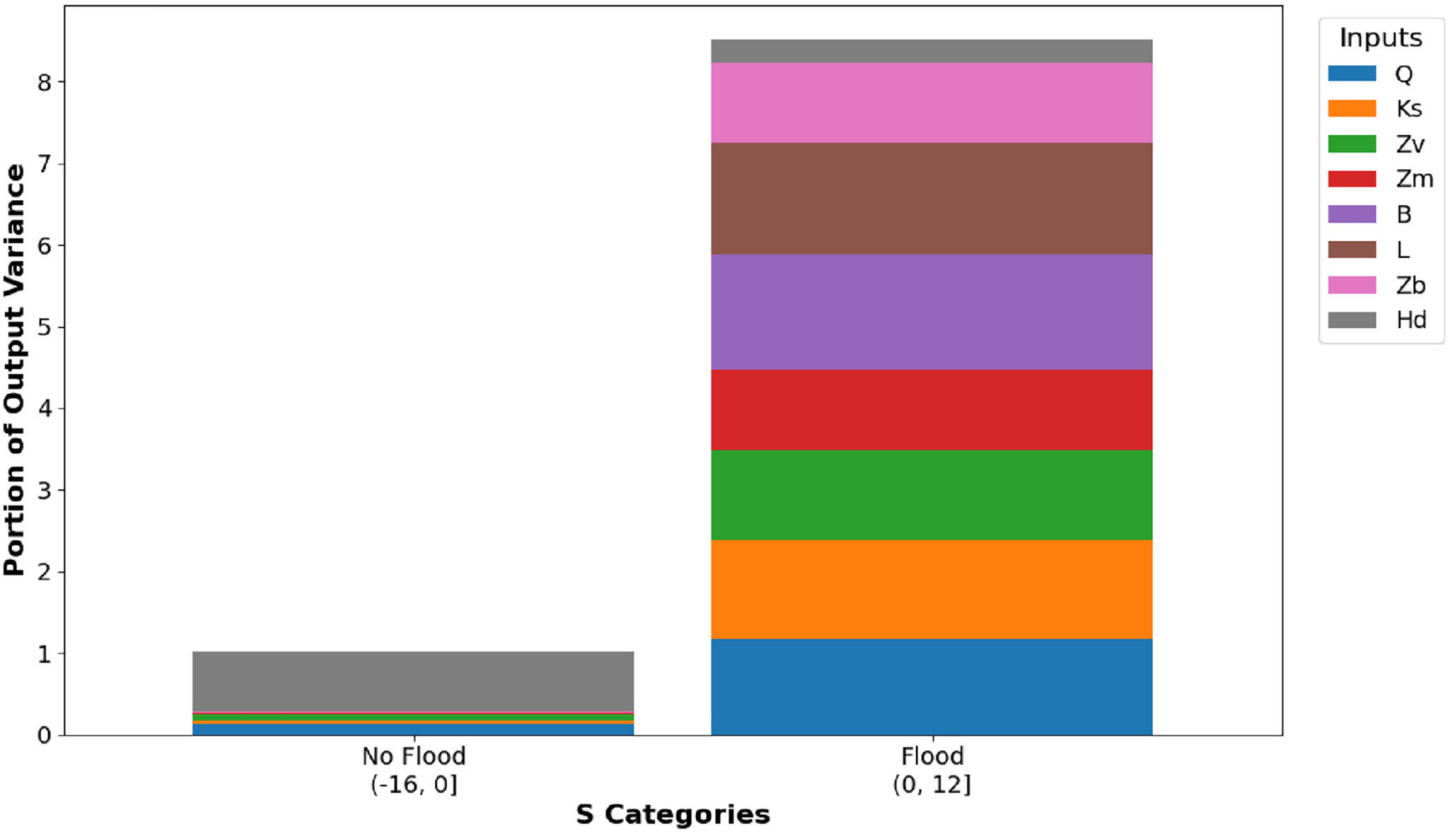
Combined sensitivity indices for the overflow S split into the no‐flood (S≤0) and flood (S>0) scenarios, based on an overall sample of $10^{5}$, of which only 23 observations correspond to the flood scenario.

The output range was divided into no‐flood (S≤0) and flood (S>0) regions to isolate the flood event and to demonstrate that the proposed approach can be applied to behaviorally defined regions as well as quantile‐based regions (Figure 20). Despite an overall sample size of $10^{5}$, only 23 observations fall within the flood region. The sum of the sensitivity indices surpassed 8 which far exceeded the theoretical value of 1 and indicated that the flood region is too small for reliable index estimation.

**FIGURE 21:**
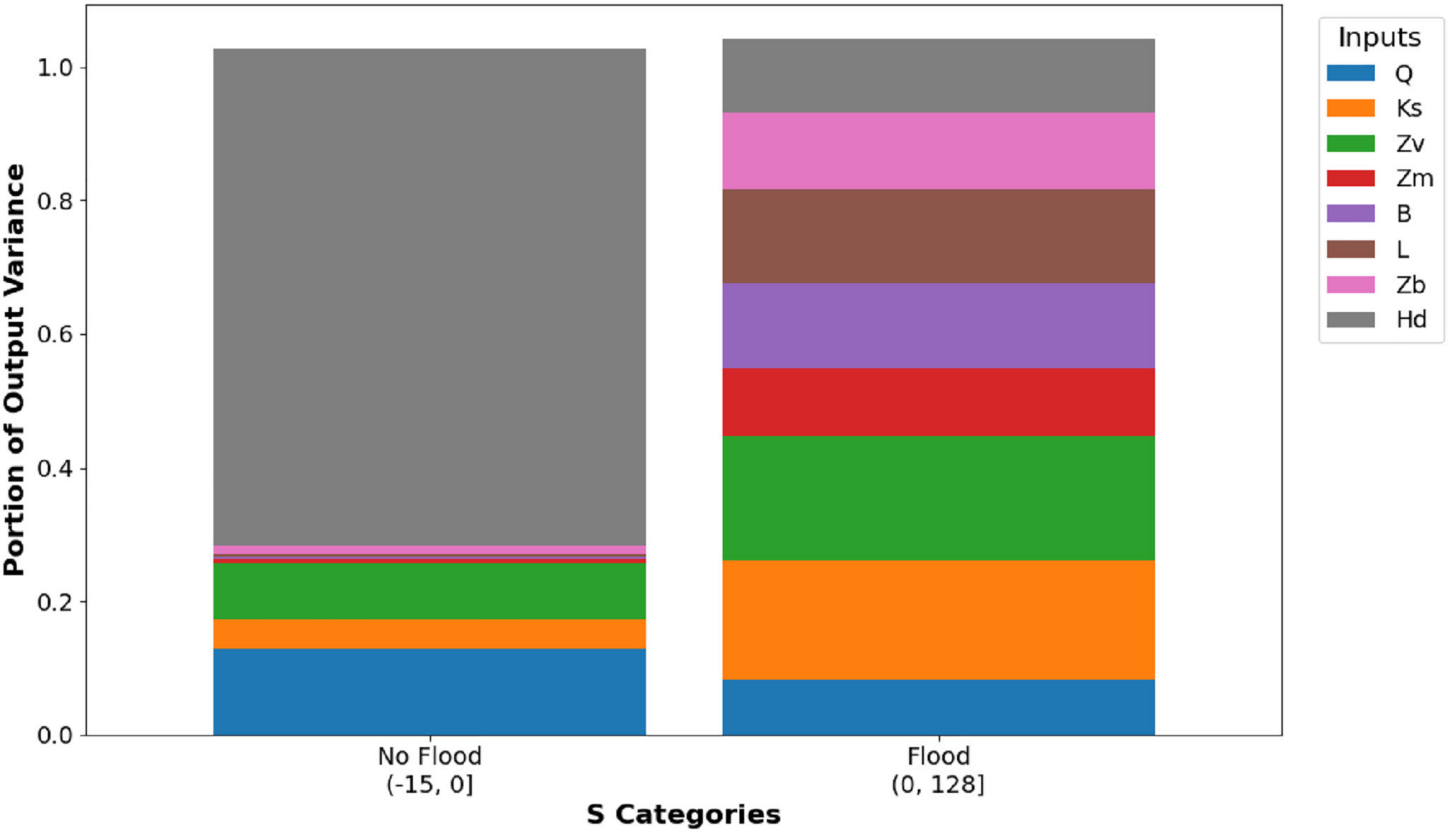
Combined sensitivity indices for the overflow S split into the no‐flood (S≤0) and flood (0>S) scenarios, based on an overall sample of $10^{6}$, of which 316 observations correspond to the flood scenario.

As a result, the sample size was increased to $10^{6}$ which subsequently produced 316 flood event observations (Figure 21). The sum of indices for both regions approached 1 which is more reliable. The flood scenario showcases fairly similar importance for all inputs except $H_{d}$ expanding the findings of quantile‐based analysis (Figure 15).

What these results imply is that the sum of the combined indices can serve as a diagnostic of whether the sample size within a region is sufficient for reliable estimation, particularly in behaviorally defined regions.

## 6 Discussion

Graphical RSA is information‐rich but quickly becomes unwieldy, as it typically requires a separate plot for each input and each output region (Baudin 2026; Hornberger and Spear 1981; Tanguang et al. 2015; Zipper et al. 2021). For instance, for the 10 output quantiles and 8 inputs used in the flood model case, a full graphical exploration of a single output would involve 80 plots, which would render any synthesis and interpretation rather challenging. Distance‐based RSA compresses these comparisons into a single summary plot (Hornberger and Spear 1981; Iwanaga et al. 2022; Pianosi et al. 2016), but its measures lack an intuitive interpretation and may not be readily comparable across continuous and categorical variables.

In contrast, the methodology presented in this paper provides a quantitative RSA expressed in familiar units, namely, portions of output variance. Represented as directly additive Shapley effects, the indices can be visualized using stacked bar charts to enable two forms of insight not available in existing RSA measures: (i) detection of regional equifinality through declines in total explained variance, and (ii) diagnosis of potentially insufficient sample sizes in behaviorally defined regions through substantial departures of the summed indices from 1.

Regional equifinality has not been considered previously in the literature and therefore represents a promising direction for further research. It also possesses practical value for determining whether a decline in an input's regional importance reflects a genuine reduction in its influence or a loss of explainability caused by multiple input combinations producing similar outputs.

The method can support several goals of regional analysis: tracing changes in sensitivity across output quantiles, identifying the inputs that distinguish behaviorally defined regions, and conditioning on inputs rather than outputs. The latter enables the analysis of multiple system regimes, exploration of relevant ranges of controllable variables as an alternative to optimization, and, for uncertain variables, can inform the fine‐tuning of their probability distributions.

## 7 Conclusions

This paper has introduced a method for regional variance‐based sensitivity analysis based on the efficient simple binning algorithm. The performance of the simple binning method on small samples is demonstrated across dozens of benchmark test functions. The usefulness of regional analysis expressed in units of output variance is illustrated through a flood model case study. In addition, the approach has unlocked a new dimension of analysis by tracing how the explainability of output variance changes across regions, thereby further supporting the analysis of complex models.

## Conflicts of Interest

The authors declare no conflicts of interest.

## Appendix A Numerical Tests

**A1‐Function**

**B1‐Function**.

**C1‐Function**.

.

**C2‐Function**.

.

**F1‐Function**.

.

**F2‐Function**.

.

**F3‐Function**.

**F4‐Function**.

**F5‐Function**.

**F6‐Function**.

**G‐Function**.

**G*‐Function(1)**.

**G*‐Function(2)**.

**G*‐Function(3)**.

**G*‐Function(4)**.

**G*‐Function(5)**.

**G*‐Function(6)**.

**Hartmann 6‐D Function**.

**Ishigami Function**.

**Kriging Model**.

**Owen Function**.

**Metafunction 1**.

**Metafunction 2**.

**Metafunction 3**.

**Metafunction 4**.

**Metafunction 5**.

**Metafunction 6**.
